# EHMTI-0037. Botulinum A toxin for treatment of chronic migraine

**DOI:** 10.1186/1129-2377-15-S1-G11

**Published:** 2014-09-18

**Authors:** L Grazzi, S Usai, G Bussone

**Affiliations:** 1 Headache Center, Neurological Institute "C. Besta", Milano, Italy

## Introduction

Chronic migraine is a common and debilitating headache syndrome.

Botulinum neurotoxin A(BoNT A), produced by the anaerobic bacterium clostridium botulinum has been recently employed for patients suffering from chronic migraine.

## Aims

BoNT A has been used in our clinical experience for treating patients referring to our headache centre and suffering from chronic migraine with medication overuse.

## Method

Forty four patients, suffering from chronic migraine with medication overuse, mean age 51.1+7.9, have been submitted to a withdrawal from medications in a day hospital setting, successively they have been treated by BoNT A injection in multiple sites according to the protocol of the PREEMPT study at the dosage of 155 U for 31 sites. Every session of local injection was repeated every three months for a period of one year.

## Results

Twenty three patients achieved the 6 months follow up. The results, recorded by the diary, evidenced a significant decrease in days of headache/month (pre 20.4±6.9 post 13.7±9.1 p<0.005) and in medication intake/month (pre 22.2±6.9 post 13.8±9.2 p<0.0005).

## Conclusion

Although these results are preliminary they led to intense efforts to evaluate analgesic properties of BoNT A and to assess their clinical applicability.

The pharmacological profile of BoNT A makes it a good candidate for migraine prevention as proposed in the PREEMPT study. Its long duration of action (3 months) makes it particularly attractive for patients who are not compliant with the daily use of preventive medications, or if they cannot tolerate them or when they are refractory to preventive medications.

No conflict of interest.

